# A Reconciliation for the Future of Psychiatry: Both Folk Psychology and Cognitive Science

**DOI:** 10.3389/fpsyt.2016.00012

**Published:** 2016-02-16

**Authors:** Daniel D. Hutto

**Affiliations:** ^1^Faculty of Law, Humanities and the Arts, School of Humanities and Social Inquiry, University of Wollongong, Wollongong, NSW, Australia

**Keywords:** philosophy of mind, narrative therapy, cognitive neuroscience, philosophy of psychiatry, philosophy of cognitive science

## Abstract

Philosophy of psychiatry faces a tough choice between two competing ways of understanding mental disorders. The folk psychology (FP) view puts our everyday normative conceptual scheme in the driver’s seat – on the assumption that it, and it only, tells us what mental disorders are (1). Opposing this, the scientific image (SI) view (2, 3) holds that our understanding of mental disorders must come, wholly and solely, from the sciences of the mind, unfettered by FP. This paper argues that the FP view is problematic because it is too limited: there is more to the mind than FP allows; hence, we must look beyond FP for properly deep and illuminating explanations of mental disorders. SI promises just this. But when cast in its standard cognitivist formulations, SI is unnecessarily and unjustifiably neurocentric. After rejecting both the FP view, in its pure form, and SI view, in its popular cognitivist renderings, this paper concludes that a more liberal version of SI can accommodate what is best in both views – once SI is so formulated and the FP view properly edited and significantly revised, the two views can be reconciled and combined to provide a sound philosophical basis for a future psychiatry.

There are more things in heaven and earth, Horatio, than are dreamt of in your philosophy.

*Hamlet* Act I, Scene 5, 167–8

## Folk Psychology Rules

How should we best understand, categorize, and treat mental disorders? A familiar answer in philosophical circles is that any approach to mental health must always operate with reference to the normative features that define our folk psychological (FP) understanding of mind. Call this the FP view of mental disorders and psychiatry. Its driving assumptions are that FP, and only FP, conceptually defines what it is to have a mind because FP, and FP alone, supplies the necessary and sufficient mark of the mental, emphasizing its essentially rational character. On the standard, narrow reading, FP plays this governing role precisely because it is understood to be the commonsense theory or conceptual scheme that reveals how mental states – typically assumed to be propositional attitudes – interact in the rational production of behavior and action.

Graham (1), a staunch spokesperson for the FP view, advances a theory of mental disorder according to which we have no choice but to make reference to reason and rationality when understanding such phenomena because such features “help to constitute and define distinctively mental activity such as believing, hoping, desiring, deciding, thinking and the like” [(1), p. 7].

The main idea behind this vision is that FP supplies the only normative standard of what minds are and how they operate. Hence, only against FP’s standard is it even possible to detect and demarcate mental disorders. Mental disorders arise when things go awry with us at some level, when in some important sense, a person fails to live up to or systematically violates the standards of rationality that characterize our everyday folk psychological ways of thinking. There may be various mental and non-mental causes of such failures. But, simply put, FP is the necessary reference point of what a normatively defined well-functioning mind looks like and what its rational characteristics are. Disordered minds, by comparison, are less than flourishing minds that fail to meet that normative standard.

Even though proponents of the FP view accept that perfect rationality is a notoriously slippery notion that evades precise analysis they insist, nonetheless, that, “rationality is essential to mindedness” [(1), p. 12]. Moreover, it is assumed that rationality is only something exhibited by whole persons and not by the operations of their subpersonal parts. Putting all of this together, in standard formulations, the FP view holds that we have no choice but to understand minds by making use of FP concepts, which apply to persons whose intentional attitudes exhibit an inherent rationality.

Thinking of intentional attitudes (beliefs, desires, and so on) as presupposing the rationality of persons makes it clear that we persons are purposive or goal directed in behavior and that how we act or behave depends on our purposes or reasons for acting [(1), p. 120].

Graham (1) dubs this the rationality-in-intentionality (RIT) thesis for short. RIT takes rationality to be the hallmark of the mental – one that sets the mental forever apart from all other kinds of phenomena, and this is what makes the mental irreducibly autonomous.^1^ The autonomy of the mental thesis can be understood in more or less realistic terms. Yet in all versions the root idea, subscribed to by all fans of the FP view, is this: propositional attitudes can only be ascribed, or only have life, when they stand in appropriate kinds of holistically and normatively defined rational relations. Mental phenomena exist if and only if the relevant forms of rationality are in place: viz. they live in the space of reasons. This is allegedly why when rationality is absent, we must switch to another scheme for understanding the relevant phenomena; in such cases, a move to non-mental concepts and explanatory schemes becomes necessary precisely because minds, properly understood, are fading or absent.

The crucial assumption of the FP view is that it is the job of philosophy of mind to reveal and articulate the essential contours of our commonsense understanding of the mind – which are assumed to be *the only* bona fide conception of mind.^2^ The standard view is that this can only be achieved by means of some kind of conceptual analysis or radical interpretation [see, e.g., Ref. (5)].

With these assumptions about the essential characteristics of minds in place, the FP view of mental disorders firmly opposes what, by its lights, is its only possible rival, a scientifically orientated, non-mentalistic FP-eliminativist approach – one that looks solely to neuroscience to discern “the best understanding of and treatment for mental disorder” [(1), p. 6]. A purely brain-based approach to mental disorders is oxymoronic from the perspective of those who hold that FP defines the mental; such an approach might tell us much about non-mental disorders of various kinds, but it could not be a starting point of inquiry into psychiatry because it misses out the mental altogether. Despite insisting on this point, fans of the FP view do not deny that neuroscience can play a part in the larger business of psychiatry. They do insist, however, that the part the brain sciences can play is always and everywhere secondary, servile, and subservient. Crucially, the FP view of psychiatry “does not relinquish the theory (of mental disorders) to, but deploys, brain science” [(1), p. 9].

Although clearly incompatible with a purely scientific, eliminativist vision of psychiatry, the FP view is compatible with making explanatory use of a range of scientific findings. The sciences of the mind have something important to add to the story so long as they take their direction from FP when it comes to understanding and classifying mental disorders on the basis of possible causes. There is no contradiction to be found in such a cooperative enterprise for those who think FP defines the mind: but this is so only as long as it is accepted that FP must always remain in the driver’s seat when coordinating any such combined efforts [(1), p. 11].^3^

The logic is straightforward. Mental disorders – on the FP view – only ever show up as disturbances within the space of reasons. Even so, there can be non-mental causes of mental disorders. We can think of such causes as arational, non-mental disorder influences that, to use Graham’s apt phrase, “gum up” “the rational works” [(1), p. 160]. Non-mental factors – the influences of brain and behavior – can interfere with and upset our rationally constrained mentality. Accordingly, non-mental factors can contribute to and help explain the occurrence of mental disorders – and this can happen even if the non-mental mechanisms in question are in perfect order and are operating just as they should.^4^

All in all, the FP view insists on a particular understanding of the explanatory relations that can hold between the mental and the non-mental when it comes to making sense of mental disorders. Neuroscience can help us to understand, for example, the condition of the unwilling addict because reference to brute arational neural mechanisms can help “*to explain* why addicts suffer from relapse in spite of themselves” [(1), p. 179, emphasis added]. Non-mental mechanisms – whether malfunctioning or not – feature in the larger story of specific mental disorders because they may explain what interferes with the rational works. Telling the right story about such non-mental influences is complicated by the fact that there can be “different hypotheses about the irruptive role of a-rational neurobiological/neurochemical mechanisms into the space of reasons” [(1), p. 178].

Speaking on behalf of the FP vision of psychiatry, Graham (1) sees no difficulty in asserting that, “even though mental disorders are not brain disorders, neuroscience helps to illuminate *their nature*” [(1), p. 13, emphasis added]. How should we understand this claim? It deserves attention for, as just noted above, the FP view holds that the essence of *mental* disorders can only be understood with reference to what occurs in the space of reasons. Accordingly, explanations that cite non-mental goings-on from outside that space can only shine a light on the nature of mental disorders if we distinguish the *essential* characteristics of the latter from explanations that tell a fuller story about their *actual* natures (or, more likely, how non-mental mechanisms make an actual but effective difference in particular cases).

This requires drawing a distinction between the *essence* of mental disorders and their *actual* natures. A standard way to do this is to take a leaf out of the analytic playbook of commonsense functionalism (6–8). Analytic functionalists hold that our everyday folk theories or practices conceptually define what minds are, fully and completely. On this score, the so-called sciences of the mind reveal nothing about the essential features of minds. *Prima facie*, this may seem extreme. However, adopting this view is entirely compatible with the idea that the sciences can discover much about how mental phenomena are, *as a matter of fact*, instantiated in the actual world – hence, the sciences can discover much about their *actual nature*. They can do so once FP tells “empirical inquiry what to look for” [(2), p. 51]. If we distinguish the essential and actual characteristics of mental phenomena and, relatedly, mental disorders, it becomes possible to understand how the sciences can assist with an understanding of the nature of mental disorders on the FP view. *Pace* Locke, on this vision, it is the scientists of mind, not the philosophers of mind, who must do the under-laboring.

## Psychiatry in the Scientific Image

The FP view of psychiatry has some fierce critics. It has been accused of presenting an unjustifiably restrictive vision of the role the sciences of mind play in mental health. Advancing the idea that psychiatry needs to be grounded entirely in the cognitive sciences, Murphy (2) is the foremost defender of the scientific image (SI) view of psychiatry. He and his friends see the FP view as unacceptable because it denies the sciences of the mind a free hand in revealing the essential character of mental phenomena. That restriction, SIers hold, results in an unwarranted fettering of psychiatric explanations and classifications [(2), p. 48, 51].

Adherents of the SI view are self-styled progressives. They insist that psychiatric explanation and nosology should not be regimented by, or beholden to, commonsense intuitions or assumptions. Psychiatric explanations, they hold, require no guidance, warrant, or mandate from FP. Proponents of the SI view insist that the future of the mental health field depends on fully embracing the sciences of the mind. The core assumption is that the sciences provide the requisite tools for a free inquiry into the nature of mental disorders. Moreover, the scientific work is to be conducted without requiring any appeal to commonsense notions of the mind, as filtered and understood through philosophy.

In place of the FP vision of psychopathology, Murphy (2) sets out his stall for a revisionary objectivism about the nature of mind. Accordingly, the bid is to discover what minds are through the development of a pragmatic and open-ended, scientifically driven conceptual framework, one that is revisable in practice and one that rests on testing out a series of empirical bets about mental phenomena.^5^ Crucially, the scientific investigations Murphy envisages would not be shackled by FP’s oversight. On the SI vision, to truly explain and classify normal and abnormal minds, we must look to our best cognitive sciences and to those alone.^6^

An immediate consequence of embracing the SI view is that it open up the scope of what we might think of as the mental and how we might think of it, quite considerably. By implication, the same goes for mental disorders. For example, given that perception is a paradigmatically mental phenomenon, it turns out that, on the SI view, blindness – however counterintuitive it may seem – counts not just as a disorder of the visual system but as a *mental* disorder [(2), p. 54, 55–57]. Murphy is happy to bite this and other, similar bullets. The justification is simple: violating a few folk intuitions is a small price to pay if going the purely SI way puts psychiatry on a “sounder footing” [(2), p. 11].

This is all very well and good as a sort of SI position statement but what justifies taking the SI path? Why suppose that SI might reveal new and deeper facts about how minds operate and how they can go wrong?^7^ Why not hold that the sciences of the mind do whatever good work they do by functioning in exactly the way the FP view says they do – viz. by supplying “the empirical application of our pre-theoretic folk concepts” [(2), p. 50]?

The most straightforward and compelling answer is that there is surely more to the mental than dreamed of by FP. This conclusion is hard, if not impossible, to resist if FP characterizes the mental wholly in terms of the propositional attitudes and how they rationally inter-relate. For on any such a rendering, there is every reason to believe that

mind has an existence and substantive character that *goes well beyond*, and is independent of our best common-sense interpretative practices. Hence knowing the truth about the mind requires *a great deal more* than informed reflection on those practices. In fact, it requires cognitive science [(9), xiv, emphases added].

A full understanding of all that is mental cannot be limited to FP characterizations alone. There are many aspects of mind – even quite ordinary, everyday ones – such as the complex ways that perceiving and acting interact – upon which FP, as construed above, has simply nothing to say. Such examples multiply. There are many forms and aspects of mentality that can only be understood by engaging in modes of inquiry that go beyond interrogating FP as traditionally conceived. FP casts no light on the properties and dynamics of basic minds [for an extensive discussion, see Ref. (10)].

Call this the “More to the Mind than FP” objection. It strikes at the core assumptions of the official FP view. Notably, however, even if the “More to the Mind than FP” objection defeats or should make us suspicious about the FP view, it does not, by itself, justify adopting the pure SI vision. For even if there is “More to the Mind than FP,” it does not follow that our understanding of minds, and by implication mental disorders, can *only* and *wholly* be supplied by the sciences of the mind.

In the end, because FP is not the whole story about minds, it will be argued that going the SI way is best – but only if SI is carefully qualified. Why so? Because FP is part of the story of the mental: arguably, important aspects of human minds can only be understood in FP terms. This can be so even if FP assumptions about the mind should not be the basis for or otherwise restrict our investigations into the fundamental nature of minds. Before attempting to show how to marry these ideas in Section “Keeping FP in the Picture,” the next two sections raise important doubts about standard cognitivist formulations of the SI view and their inherent neurocentrism.

## A Certain Irony

The SI view is open to understanding the mind in new ways that go beyond FP. Despite the essential openness of the SI view, some of its most prominent proponents have tried to foreclose on certain possibilities. Based on assumptions about what the best explanations in the cognitive sciences will look like, some campaign for a neuro-based cognitivist version of the SI view. For example, under SI’s auspices, Murphy offers a defense of the idea that, “psychiatry is a branch of medicine dedicated to uncovering the *neurological basis* of disease entities” [(2), p. 10, emphasis added].^8^ For him, going the SI way paves the way for adopting the medical model of psychiatry such that the work of psychiatry becomes that of tracing “abnormalities in behavior and cognition to specific *causal factors that are realized in brain* tissue” [(2), p. 13, emphasis added].

In Murphy’s mind, adoption of the SI view leads naturally to firmly recommending a merger of psychiatry and clinical neuropsychology.^9^ He is supremely confident that a purely neuro-based approach will dominate the future of psychiatry. This is evinced by his commitment to neurocomputationalism, input–output functionalism, modules, and so on. But why assume that the brain’s the thing? Retort: Who seriously doubts it? Murphy tells us that, “After all, *everyone knows that* psychological phenomena, like all human behavior, are *rooted in* brain processes” [(2), p. 9, emphasis added]. How should we interpret the “everyone knows that” operator in this statement and what epistemic backing does it have? There are several possibilities.

The first is to go “Folk Analytic.” Perhaps we can appeal to folk intuitions about the mind to justify talk about what everyone knows about the brain basis of minds. Clearly, this is a non-starter for SIers. The SI view precludes making any appeal to hypothesized folk theories and the intuitions they sponsor in order to explain the epistemic credentials of “everyone knows” talk. Folk intuitions can give no backing to SI friendly claims about what everyone knows; hence, they cannot help justify the claim that cognition is wholly caused by and realized in the brain as opposed to having a wider and non-exclusively neural basis. Put simply, to adopt the SI view of psychiatry is to forego making appeals to folk intuitions in order to defend claims about “what everyone knows” about the mental. Call that Murphy’s Law. Murphy too must abide by it.

The second way to go might be to appeal to consensus in this matter in the philosophy of cognitive science. Classical cognitivist approaches to cognition promote a brainbound account of cognitive processes by adopting a representationalist and internalist account of the vehicles of cognition. If everyone in the field agrees that cognition is always and everywhere content involving and that the vehicles of mental content are neural, then this would justify claiming that “everyone knows” neurocentrism to be true. The best cognitive explanations of behavior can, in effect, “throw away the world” and focus solely, and solipsistically, on the properties that supervene on the current internal neural states of cognizers (11–14).

SIers would be justified in saying that psychiatry ought to take an exclusive interest in brains if classical cognitivism were true. The trouble, for Murphy and followers, is that classical cognitivism may not be true, and – as things stand – it is far from a safe bet to assume that it is. More to the point, looking at the state of the philosophy of cognitive science, it can be safely said that classical cognitivism is *not known* to be true. There are deep-seated, on-going philosophical debates about the character of cognition and the reach of cognitive processes – and these debates are far from being conclusively settled.

Murphy is well aware of these debates and their import. His official word on the matter in 2006 was to note that, “Some pictures of the mind stress embodiment very heavily … Others prescind from details of our embodiment to stress a more purely computational theory of the mental … what counts as the mental depends in part on who is right in these debates” [(2), p. 64]. Despite this acknowledgment, Murphy thinks there is really no doubt that the sciences of mind will stick to providing explanations in terms of brain-based, semantic representations. This he takes to be a settled issue – even if, in the end, the hypothesized brain-based representations in question turn out not to have contentful properties of the FP sort.^10^

However, what exactly are the defining properties of representations, as defined solely by the sciences of the mind, without any reference to the kinds of content understood by FP? How should we understand the disagreements between representationalist and non-representationalist if we do not appeal to some notion of content as supplied by FP or some other agreed upon non-FP theory (15)? And without agreement about the defining properties of representations understood in non-FP terms – which might be supplied if we had a well-developed non-FP theory of content – how are we to decide where the boundaries of mind and cognition lie? How are we to determine whether – in the end – the best explanations of sciences of the mind will be given in terms of “inputs” and “outputs” that are purely neural as opposed to involving extraneural factors too (16)?

Against this backdrop, Murphy’s confidence in an exclusively representationalist and neuro-focused future for psychiatry will seem, at best, premature – and at worst, it will look like a groundless pledge of allegiance. For the fact is there is no agreement in the philosophy of cognitive science that supports the idea that everyone knows – at least, not yet – that psychological phenomena are rooted or realized exclusively in brain processes.

But wait. Surely, we are looking for consensus in the wrong place. The fact that philosophers – of the mind or otherwise – disagree about important topics is hardly news. Perhaps there is yet another, more properly scientific consensus that we can appeal to in order to make good on the “everyone knows” claim. Doesn’t a quick glance at the current agreement in the theoretical commitments of actual scientists of the mind secure its truth? The great bulk of scientists of the mind do talk of neural and mental representations in free and easy ways these days. Does it follow that they are committed to a cognitivist take on mental representations of the sort described above – one that would justify neurocentrism? Establishing that would require serious and detailed interpretative work: it would need to be shown that the representational talk of scientists has all of the relevant commitments and that it is more than nominally unified. It is far from obvious that this is the case. One major problem is that no unified theory of representation currently exists. Worse still, if we look at the current state of cognitive science there does not seem to be a single, settled story to tell about which theoretical tools – representational or non-representational – are primary or the best ones to use when it comes to understanding cognition and explaining intelligent activity. We seem to be living in a mixed economy. If this is right, then there is not an existing scientific consensus SIers can point to in order to justify the claim that “everyone knows” cognition to be brainbound.

On top of this, even if such a current consensus did exist – even if all good cognitive scientists turned out to be representationalists in the relevant sense – more work would be needed in order to determine whether the entities and properties they posit now will stand the test of time. It is always possible that even if all cognitive scientists are currently committed to neural representations still, it might turn out that something with different properties will best explain the relevant phenomena. Cognitive science is, after all, an unfinished business. Hence, even if today’s scientists did have common commitments that would justify adopting neurocentrism, we might still worry that any such contingent fact would not provide a secure basis for making firm predictions about the future of psychiatry. The official story is that not long ago cognitivism replaced old school behaviorism, right? Science is shifty – but in a good way. The SI view should surely embrace that.

At this stage of the game, there seems to be no obvious justification for fans of the SI view to reject the idea that psychiatry might look beyond the brain when it comes to understanding, explaining, and treating psychopathological disorders. Indeed, there are positive reasons for thinking that it is fruitful to look beyond the brain when it comes to understanding mental phenomena (16). Notably, since looking beyond the brain does not entail ignoring the brain, adopting such a liberal SI view is perfectly in line with a modified version of Murphy’s assertion that “we are animals with a biology including a brain that is [part of] the foundation of our mental life” [(2), p. 10].

Still, it might be thought that the foregoing liberal assessment is too blithe, quick, and programmatic. Aren’t there good grounds for thinking that cognitive science will remain deeply committed to cognitivism and neurocentrism, even if in the final reckoning, it deviates in some matters of detail from the classical versions of those views? Aren’t there special reasons for favoring cognitivism – reasons that we can identify here and now – that would justify neurocentrism and thus rule out more radical and extensive possibilities for understanding the nature and extent of cognition. That seems to be the line of several prominent defenders of cognitivist variants of the SI view (2, 3).

## Cognitive Bridge Work?

In defending their predictions about the rightful dominance of an SI-based medical model, Murphy and Smart (17) make it clear that this future is to be secured by *cognitive* neuroscience and not merely some brutal brain science. What makes cognitive neuroscience special, they maintain, is that it posits subpersonal information-processing mechanisms that are at once both causal–mechanical and intentional in character. It is because it blends the cognitive with the neural that cognitive neuroscience has unique explanatory power: it, alone, allows for an integrated scientific story to be told about minds.

Looking exclusively at what goes on in brains is apparently justified because of the depth and unity cognitive neuroscientific explanations can provide. Gerrans (3) makes this case in great detail.^11^ He argues that cognitive neuroscience understands, “persons as complex, hierarchically-organized information-processing systems implemented in neural wetware” [(3), p. 16].^12^ Seeing persons as brain based, in turn, allegedly confers peculiar advantages because it puts us in a position, for example, to “show how *facts* identified and explained by disciplines operating at ‘levels’ such as molecular neurobiology or neuroanatomy *can explain* psychological and phenomenological level *facts* that give delusion its clinical profile” [(3), p. 20, emphases added].^13^ What makes having a cognitive theory pitched at the subpersonal information-processing level so uniquely valuable is that it is needed to “*bridge the gap* between neurobiological and personal level explanation” [(3), p. 21, emphasis added].

Integrated explanations of the promised kind are said to be unavailable, in principle, to the isolationist FP view: this is precisely because to adopt the latter’s “space of reasons” idea enforces an absolute distinction between the intentional and the mechanical.

To see what makes cognitive theory so appealing, it is worth getting clear about what exactly the FP view allegedly cannot do. Gerrans (3) accuses its proponents of operating with a disunified framework – one in which mechanisms are assumed to make only a causal difference to cognitive goings-on in a way that debars them from being properly explanatory (p. 15, 20). For example, Gerrans characterizes the FP view as being committed to the idea that organic damage might “*play a causal role* in introducing the drastic change in psychological structure but *plays no explanatory role*” [(3), p. 27, emphases added]. Does it make sense to think the explanatory space could carve up in the way Gerrans suggests? Can we distinguish between something’s playing a merely causal versus a properly explanatory role? How should we understand this distinction?

As discussed in Section “Folk Psychology Rules,” proponents of the FP view clearly allow that mental phenomena can be explained by what goes on in non-mental mechanisms. The FP view may be limited in that it is not interested in, or simply fails to provide, very detailed stories about the non-mental causal contributions of implementation mechanisms in information-processing terms. But it cannot be faulted for ruling out, or making it impossible to tell, such deeper explanatory stories. So this alone cannot be what makes its rival, the cognitivist view, special.^14^

Apparently, what makes cognitive theory special is that it brings something else – something quite unique – to the table. It regards the mind as a complex information-processing system – one that is organized in a hierarchical way, with a variety of interacting processes playing specific roles and where some of these diverse processes are responsible for the supervision of others in the system. Understanding the mind through the lens of cognitive theory allegedly provides a peculiar sort of intelligibility – one that allows theorists to go beyond the telling of merely “difference making” causal stories. The cognitive theory allows us to see how everything fits together in a systematic way; it bridges the gaps and enables explanations at many different scales and levels to be integrated by detailing how information flows from level to level and what role particular processes play in the wider cognitive economy [(3), p. 48, see also 32, 53, 79, 103].

From this vantage point, it is easy to see the attraction of having a broader vision of the mind that seeks to understand the roles played by various forms of cognitive activity, how various aspects of mind relate to and interact with one another, and how specific disturbances in those relations and interactions can lead to mental disorders with signature profiles.

This much is welcome. Yet friends of cognitivist SI, such as Gerrans (3), go further than this: they suggest that only cognitive neuroscience has what it takes to do the required integrating work. As Gerrans (3) says, “the *essential idea* of cognitive neuropsychiatry is that without a cognitive theory the problem identified by autonomy theorists … *cannot be solved*. The gap between neurobiology and psychology will be *unbridgeable*” [(3), p. 36]. Hence, “there *must be* an explanatory relationship between neuroscience and folk psychology” [(3), p. 33, emphasis added]. These are very strong, philosophically “musty” claims – and they are not self-evidently true.^15^ We might well doubt that cognitive neuroscience *per se* is best placed to provide the desired integrating theoretical vision, especially in light of the concerns raised about Murphy’s neurocentrism in the previous section.

What might persuade us that a brain-based cognitive theory is necessary to bridge the putative gaps? Allegedly, that cognitive neuroscience supplies special means for understanding the links between various mental phenomena. It can do so, again allegedly, precisely because it endorses a vision of the neurally housed mind “organised as a hierarchical system … which *uses representations* of the world and its own states to control behaviour” [(3), p. 47, emphasis added]. The claim is that cognitive theory posits neural representations that perform a variety of cognitive tasks and that once we understand how information flows between such representations, we will be in a position to provide complete and satisfying explanations of mental disorders in ways which make the links between subpersonal to personal-level cognitive phenomena intelligible. Thus, Gerrans (3) observes of this general strategy that, ultimately, supplying the correct account of what drives specific delusions requires accounting for “the way the brain encodes information acquired in experience and then reconstructs representations of that information when subsequently cued” (p. 33).

It seems that the central posits of cognitive theory – information and representation – provide the perfect theoretical glue for integrated explanations. Cognitive neuroscience promises to show how there can be relevant connections between various cognitive activities in a way that does not just cite correlations or brute causal relations. Instead, cognitive neuroscience alone proves to be genuinely explanatory of mental disorders because it alone *makes intelligible* multilevel interactions across various scales and levels.

Allegedly, cognitive neuroscience alone can achieve this feat because it is wedded to a representational theory of mind that assumes cognition to be at root both mechanical and intentional. Importantly, cognitive theory seems to provide us a new mark of the mental – not “rationality-in-intentionality” cast as person-level phenomena, to be sure. Instead it offers us a “content-in-intentionality” or CIT mark of the mental.^16^ For those who accept something like CIT, even though the cognitive is regarded as quite a mixed bag that reaches across the so-called subpersonal and personal levels it is also united by the intelligible relations that are instantiated through the processing of informational and representational content in ways that define minds.

Some philosophers hold that cognitive scientists are committed to essentially characterizing minds in information-processing terms. This is, of course, not news. We frequently hear that

cognitive science … has as its subject matter capacities like memory, perception, attention, language processing and reasoning. The concepts that cognitive sciences *take to be essential* for understanding their domain include information, representations, and algorithms [(19), p. 74, emphasis added].

Let us suppose, for the sake of argument, that Shapiro (19) is right in thinking that working cognitive scientists take themselves to use and need these kinds of conceptual tools. Would this help fans of the cognitivist SI view to justify the claim that cognitive neuroscience operates with unique explanatory tools that give it special gap-closing powers?

Would assuming CIT make cognitive neuroscience ideally well placed to provide gap-bridging solutions? One reason for thinking so is that CIT seems to imply the existence of something like a neurally based space of reasons. To posit a space of reasons mark II would be to assume that there exists a cognitive level at which various mental phenomena do not just brutally interact but intelligibly inter-relate because they communicate by trafficking in contentful information and representations. Content would, on this picture, be the shared common coin traded by all cognitive phenomena. The CIT picture seems to make it possible to understand cognitive relations in explanatorily illuminating ways that do not reduce to the giving of merely brutal, causal explanations.

Let us imagine that cognitive neuroscience posits a neural space of reasons, ala CIT, and embraces internalism about the vehicles of various mental contents. If so (assuming the above analysis is correct), it would follow that cognitive neuroscience would have utterly special resources for bridging the sort of gaps of which Gerrans (3) speaks. All that would have to be done to seal the deal would be to show in detail how the cognitive neuroscience, as imagined above, could use those resources to in fact close such gaps. Doing all of this would be an effective way of motivating an exclusively neurocentric version of the SI view.

Before assessing whether cognitive neuroscience, so construed, really has what it takes to close the said gaps, it is important to be clear about the source of the alleged need to do so. Notably, if there are any such explanatory gaps to bridge, then the need to bridge them is motivated by purely philosophical, not scientific, considerations. Without doubt, scientists and psychiatrists seek rich explanations of the roots of mental disorders. Providing such explanations would require going beyond FP and delving deeply into the sciences of the mind. However, crucially, providing such explanations is not the same as, nor does it require, bridging the putative intelligibility gaps – those that hold, e.g., between neurobiology and folk psychology and with which Gerrans (3) is concerned. Seen in this light, it becomes clear that Gerrans (3) seeks to motivate an exclusively cognitive neuroscientific take on SI by getting us to take seriously the need to address explanatory requirements of a distinctively philosophical kind.

The great irony is that elsewhere SIers reject the need to address such intelligibility demands as illegitimate. Compare the alleged need to make sense of the interactions between cognitive phenomena across levels by appeal to representational contents with the alleged need to make sense of the connections that hold between propositional attitudes in terms of rationality. If Gerrans (3) is right, cognitive theory can help us to make intelligible how various subpersonal cognitive phenomena inter-relate. How might it do this? By rendering the relations between cognitive phenomena intelligible. How? Not in RIT terms that explain how personal-level propositional attitudes relate rationally, to be sure, but in CIT terms that explain how neural representations relate contentfully.

It should now be easy to see why it would be a problem for SIers to advance this type of line. Any attempt to motivate a neurocentric cognitivist SI view by arguing that cognitive neuroscience alone can bridge otherwise unintelligible explanatory gaps requires being sensitive to the very sort of philosophical concerns that the SI view itself casts into doubt. Must there be some common feature (if not rationality then content) that is shared by all mental phenomena and which unifies them and explains how they intelligibly inter-relate? SIers say “No”: They question the demand that “personal-level phenomena can only be explained in terms of other personal-level phenomena” [(3), p. 21]. This being the case, surely, we are also well within our rights to question whether there is a legitimate need for a unifying cognitive theory that makes intelligible how various cognitive phenomena intelligibly inter-relate in special, more-than-merely causal ways. As the old proverb reminds us, what’s sauce for the goose is sauce for the gander.

And there is something else to consider. We might doubt that on close scrutiny appeals to information and representation could play the unifying and integrating roles that would satisfy the identified gap-bridging needs, if we were to take such needs seriously. The fact is that apart from bearing the names “cognitive,” “representational,” or “informational” nothing in so-called current cognitive theory deeply unifies all the various cognitive phenomena in terms of their importantly and interestingly diverse properties or roles.

Consider Gerran’s claim that, “a scientist explaining some discrepant evidence *is doing the same thing* as the oculomotor system controlling the trajectory of a limb” [(3), pp. 46–7, emphasis added]. Is this credible? Undoubtedly, there may be some mileage in taking this route and drawing loose analogies for certain purposes. But a developed theory would be needed to back up any such claim if taken in a serious and literal way.^17^

To illustrate the point consider what Gerrans (3) has to say about the activation-information-mode (AIM) model of dreaming, which focuses on the flow of information within and between components of a control hierarchy. In discussing that model, he holds that the “*intrinsic cognitive properties* of these components *are preserved through transitions from mode to mode*. What changes are the interactions between these components” [(3), p. 79, emphases added].

Usually, in this sort of context, cognitive theorists upgrade talk of the flow of information to talk of the flow of informational content. Content, they hold, is what survives changes in mode and process. Canonically, the content of a mental state is determined by what it is about and how it represents the world to be. Now, if information processing literally involves the trading of contents that would make it easier to justify claiming that scientists and information-processing systems basically do the same cognitive work. Moreover, if content were the common coin that is always traded in some form, everywhere in the cognitive economy, it would be clear why there would have to be, and how there could be, intelligible relations holding between the many and various cognitive phenomena.

The trouble with this gambit is that it raises a host of unanswered questions. Just what is informational content anyway? What intrinsic cognitive properties does it have? Where does it get them? How can content be preserved through changes? How can it make a difference to cognition? How does it relate to representational content? Is it a kind of objective commodity? Does it make sense to say that we can take different perspectives – e.g., subjective and objective – toward it [as Ref. (3) appears to assume – see, e.g., p. 17]?

Cognitive theorists can avoid these tricky questions by sticking to an understanding of information in scientifically respectable terms – those of covariance and correspondence. That is perfectly fine, but then it is difficult to justify claims that basic information processing is content involving in a way, which would license drawing a strong analogy with the theoretical activity of scientists.

This is just one example of a disunity objection to the integrationist picture. To make a full dress case against such a vision would require a much longer discussion [(10), esp. ch. 4]. For our purposes, it suffices to note that anyone offering a bridge building, unifying cognitive theory must answer the sorts of questions raised above. *Prima facie*, it seems they will only be able to do so with the backing of a well-developed naturalistic theory of content.

To highlight why such a theory is needed, consider a different set of cases. In many of the explanations that Gerrans (3) offers of delusions the “felt” aspect of the phenomenon in question turns out to be a pivotal factor. The phenomenological and emotionally charged aspects of our experience apparently matter to and help explain some of the strong tendencies we have when responding to, interpreting, and accounting for our situations. Yet, as is notoriously well known, we currently lack anything like a workable theory that shows how we are to understand such qualitative phenomena in purely information processing or representational terms. Once again, it looks like disunity rather than unity is the word of the day.

Things are even more puzzling if we consider the roles imaginings are meant to play in the integrative explanations on offer by cognitive theorists, such as Gerrans (3). For example, he holds that simulative activity generates imaginings that can be incorporated in a wider cognitive economy. By this, he means that imaginings can be the basis for action (including mental action). Despite the fact that imaginings are influential and we often act on them, they are cognitively interesting and distinct because they lack many of the properties of canonical propositional attitudes, such as belief [(3), p. 18].

On this score Gerrans (3) tells us that

Imagination uses the mind’s cognitive resources, such as perceptual, doxastic and emotional processing to create simulations. It thus inherits the intentional structure of these counterpart processes. However *qua* simulations *imaginative states do not have congruence conditions*. [(3), p. 105].

The basic claim, which is plausible enough is that imagination deploys specialized neural circuitry to “construct and manipulate *representations which have representational contents but no congruence conditions*” [(3), p. 114, emphasis added]. Gerrans (3) is concerned to show that simulative imagining can figure in and make a difference to one’s thinking without the content of such imaginings being believed.

Yet, since most theorists hold that mental content requires some kind of correctness or congruence condition, it is puzzling in what sense imaginations can be said to have representational content if they lack such conditions altogether in the way Gerrans (3) proposes. What remains if you subtract congruence conditions from a mental representation? Gerrans’s (3) answer is intentional structure. But it is not clear what exactly puts the intentionality in this structure for cognitivists if not the existence of mental representations with congruence conditions.^18^

Let us be clear. A simulative account of imaginings is attractive for many reasons [(23), ch. 4]. However, it is far from clear that imaginings without congruence conditions are best understood as any kind of mental representation for precisely the reasons stated above (20, 22). But even if this proves possible it would remain unclear how a simulative account of the imagination that emphasized the lack of congruence conditions could contribute to a unified cognitive theory of minds.

Our capacity for producing narratives – often quite spectacular ones – is yet another place in which it is important to recognize that interesting forms of cognition have special properties that break the standard representationalist mold. Gerrans (3) proposes that particular forms of delusional thinking arise from signature breakdowns in the usual interactions between cognitive systems. These breakdowns in turn prompt patterns of default thinking that take the form of experientially charged imaginative episodes. Default thoughts of this stripe provide raw material that can be woven together into what are, for those in the grip of a delusion, spectacular and hypersalient narratives. Importantly, such default thoughts “are subjectively adequate responses to experience constructed as narrative elements or fragments” [(3), p. 101].

The basic idea is that when operating in the default mode, we assemble first pass, coherent stories. Yet even when these stories are internally coherent, they are not always subjected to critical epistemic scrutiny. According to Gerrans (3) when unsupervised by decontextualized systems, the products of default thinking are not scrutinized for consistency or veridicality; they are not evaluated against “competing narratives for accuracy or utility” [(3), p. 77].

This is hardly surprising since the great bulk of narratives do not aim at truth. Although narratives all share certain basic structural properties, we must look to the contexts in which we use a given narrative in order to determine its semantic properties. Thus, as Goldie (24) points out “Fictional narratives do not aspire to be true, whereas real life narratives do. A narrative is fictional not in virtue of its content being false, but in virtue of its being narrated, and read or heard, *as part of a practice of a special sort*” (pp. 152–3, emphasis added). Thus fictional narratives, offered up as fictions, invite “the audience to imagine or make believe that what is being narrated actually happened, even when it is known that it did not. Thus the question of reference and of truth simply does not arise within the ‘fictive stance’” [(24), pp. 152–3]. For these reasons, Goldie concludes that, “reference and truth have no application in fiction, but do have application in historical and everyday explanation” [(24), p. 154]. Different kinds of narratives exhibit different kinds of semantic properties, and we understand these differences if we are alert to the roles that these different kinds of narratives play in our lives and thinking.

A crucial contrast becomes evident if we compare the uncritical use of narratives that do not aim at truth with the intense critical scrutiny of beliefs and claims that do. In the most serious cases, the latter are subject to the norms of scientific testing, where we seek to fix what we believe only “according to standards of consistency and empirical adequacy” [(3), p. 13]. Put simply, some forms of cognition do have representational contents and do play roles in our cognitive economy that make them subject to epistemic norms which simply do not apply to other forms of cognition. Other forms of cognition lack these features. Our so-called default thoughts – those generated when our minds are wandering or in screensaver mode are a prime example. They do not involve any “attempt to confirm an empirical hypothesis” [(3), p. 76].

Although the above analysis only scratches the surface, the important thing to note is that the detailed explanations Gerrans (3) offers of the complexities of delusional thinking gain their power by focusing on the way diverse cognitive phenomena (e.g., feeling, imagining, and narrating) interact in virtue of their special cognitive roles and properties. Contrariwise, these explanations gain nothing from making the additional cognitivist assumption that all mental phenomena are united because they are, somehow, representational in character.

To tell a convincing explanatory story about minds and how they can become disturbed in particular ways, we need to recognize the important diversity of mental phenomena rather than insisting on a cognitivist account that downplays those differences in favor of fulfilling a philosophically motivated demand for unity. We can relinquish CIT and its problematic intelligibility requirement, recasting the integrating cognitive theory in far less ideologically demanding ways than do the friends of cognitivist SI. This does not mean we should give up on understanding how various mental phenomena interact or that we should not seek to understand the roles they play in the larger cognitive economy. It simply means that we can make sense of the relevant interactions and relations between mental and other phenomena without insisting that informational and representational content are needed to account for the intelligibility of such relations.

Only if we fully free ourselves from the constraints of FP-based philosophical suppositions about what is necessary for something to count as a properly cognitive phenomena does it become possible to concoct accounts of cognition that are truly unconstrained by FP thinking about the basic nature of minds. Interestingly, radically enactivist approaches that lay stress on the importance of interactions over contentful representations as the common coin of the cognitive looks well placed to pick up the explanatory burden (10). This is especially so if it is accepted that “what needs to be explained here is not just the causal interactions among neurons but *the way those interactions* enable cognitive processes and experiences” [(3), p. 30].^19^

## Keeping FP in the Picture

Only once the siren songs of an exclusively brain-focused future vision for psychiatry are silenced can the ground for a suitably open-minded and philosophically uncontaminated rendering of the SI view be laid. This closing section shows that when modestly formulated in the way suggested above, the SI view can make peace with an unimperialistic vision of FP.

Consider, once again, Gerrans’s (3) plausible suggestion that narrative-based and theory-based explanations differ in important ways because they answer to different epistemic standards. Thus, to understand the delusional mind requires understanding how these modes of cognition interact or fail to interact in particular conditions.

Let us assume that Gerrans’s (3) answer is along the right lines. Let us assume, for example, that those under the sway of specific delusions do indeed construct stories as opposed to rationally evaluated beliefs in order to make sense of such episodes. We might wonder, assuming they are not natural-born narrators, how they come to be able to weave such stories? We might be interested to know why a given kind or genre of story rather than another is more compelling to some populations rather than another? Or, why – upon experiencing an underlying mismatch between what-is-felt and what-was- anticipated-would have-and-should-have-been-felt – the narratives of deluded people unfold in one standard variant rather than another. The thing to notice is that in order to explain and understand key features of delusional narratives and the narrative practices that enable their generation requires looking at socioculturally and not purely neural factors [for extended arguments along these lines, see (23, 25–27)]. This is especially the case when it comes to understanding the distinctive kinds of norms relevant to the sorts of cognitive activity that differentiate narrative from scientific practices.

The point is that understanding the relevant norms requires looking beyond the brain (27). Only outside the skull of individuals do we find what we need for making sense of normative features of the cognitive phenomena that need explaining. Yet it is also when we look to certain public practices that we come by the resources for adopting a softer take on FP and its role in therapy. Certain kinds of treatment urge us to make best use of tools already available within cultures – such as incorporating traditional narrative practices into therapy – in order to respond to those in need.

There are compelling reasons to agree with Gerrans (3) that our foremost ways of making sense of ourselves and others are grounded in explanations that are not theoretical but narratively based. Such explanations function, primarily, as normalizing explanations. In giving them, any of a number of explanatorily relevant factors might be cited (e.g., facets of X’s character, X’s mood, X’s larger projects, the content of this or that propositional attitude of X, and so on). Crucially, like historical explanations, these folk psychological explanations are not general and abstract but take the form of narratives that emphasize details that are personal and particular.

Consider an idealized test case. Imagine a person suffering from a psychiatric condition that is brought on by a cascade of factors rooted in neural causes. Imagine that the condition can be wholly and successfully addressed by a perfectly targeted neuroscientific intervention. Even in this imagined case – one that best favors a purely neurocentric vision of psychiatry in terms of diagnosis explanation, and treatment – it is plausible there would be a need for the person to achieve a rehabilitating self-understanding. Graham (1) captures this point when he says:

to mend or heal from a disorder in a self-respecting and dignified manner requires discovering a positive or purposeful place for past and present episodes of disorder in the … course of a person’s life … [this] often consists of dealing with conflicting interpretations of one’s past … [(1), p. 14].

The take-home lesson is that even in ideal cases in which targeted neural inventions might wholly relieve specific conditions we should not typically expect psychiatric therapy to boil down to a simple business of eradicating “disease” in the way a narrowly construed medical model can suggest.

Murphy (2) appears prepared to acknowledge that there is a need for psychiatry to go beyond the brain, at least in some cases. In this vein, he states clearly that, “there are important roles for non-scientific thinking about the methods of psychiatry” [(2), p. 47].^20^

Importantly, even for those who press for a more thorough brain-based vision of psychiatry, there need be no conflict between endorsing both an SI view of the field and recognizing the need and importance of non-scientifically focused therapies.^21^ When it comes to therapy, a cautious pluralism seems to be the appropriate stance: it appears we need a variety of approaches if we are to improve the situation of individuals. Individual therapeutic requirements need to be assessed on a case-by-case basis. What matters is that a pluralist approach is always possible – and typically desirable – when it comes to treatment. As Murphy (2) rightly stresses, “Even if we have established that a symptom is best explained in terms of one main causal factor, such as neurotransmitter abnormality, it does not follow that treatment must be directed at directly manipulating that causal factor” [(2), p. 369].

This is all well and good, but Murphy (2) is almost completely silent about which non-scientific approaches and forms to therapy might be usefully brought to bear. And it is here that folk psychological narrative practices are likely to play a central role. This is because narratives are the familiar, everyday medium through which most of us readily evaluate and reflect upon our reasons, attitudes, and situations (24). Reviewing and recasting our narratives, with the assistance of others, is not only a way of making sense of our lives in new and fresh ways it can open up possibilities for living them differently^22^. Narrative practices afford such new possibilities precisely because they provide a means for thinking afresh about “who we are” based on richer understandings of our peculiar situations by revisiting our possible pasts and reimagining our possible futures.

Understanding FP as a kind of narrative practice in this way connects perfectly with the ambitions of narratively based therapies that seek to use so-called “talking cures” to empower people in the construction of a viable “future trajectory rather than achieving past accuracy” [(1), p. 14]. FP, as a special kind of narrative practice, is a possible object of philosophical and scientific study in a way that is wholly compatible with the modest rendering of the SI view argued for in the previous section. The views are compatible because FP, construed as a narrative practice, is not to be understood as a general theory embedded in that practice from which a philosophically discernable mark of the mental that defined mental disorders is to be sourced.^23^

## Conclusion

There are excellent reasons to resist a forced choice between standard format FP and SI views of psychiatry. On the one hand, in its original variant, the FP view attempts to provide a definitive mark of all that is properly mental, which is imperialistic and isolationist. On the other hand, the SI view, at least when formulated in its popular cognitivist version, is unjustifiably and potentially unhelpfully overly narrow and neurocentric. Consequently, adopting either of these views of psychiatry in their standard forms threatens to leave us with an ideological vision of psychiatry’s future that is too extreme and too limited. A better way forward is to salvage what is best from heavily edited versions of the familiar versions of the FP and SI views on the market, combining what remains to best effect.

Ultimately, the arguments presented here have been pitched at a quite general level, whereas to make good on this plan for reconciliation in a wholly convincing manner requires more detailed philosophical work on case studies in ways, which it has not been possible to provide in this paper. But if the above arguments are sound, then the ambitions of this paper will have been realized and the ground will have been laid for those future, follow-up endeavors.

## Author Contributions

The author confirms being the sole contributor of this work and approved it for publication.

## Conflict of Interest Statement

The author declares that the research was conducted in the absence of any commercial or financial relationships that could be construed as a potential conflict of interest.

## Footnotes

^1^The FP view endorses the irreducibility claim about the mind, which Davidson championed long ago: “The reason mental concepts cannot be reduced to physical concepts is the *normative* character of mental concepts” [(4), p. 46].

^2^Those who take the autonomy thesis seriously – whether in strongly realist or more interpretationist renderings – maintain that an FP understanding of mind plays an absolutely essential role in defining what minds are and, hence derivatively, what *mental* disorders are. Accordingly, “the mind *qua* mind puts its inscription on the sources of a disorder. We cannot recognize a mental disorder without uncovering that mark” [(1), p. 11]. It is because of the need for benchmarking against the mark of the mental – which can only be done *via* FP – that “no conceptually regimented and normatively informed theory of mental disorder can be devised without taking philosophy of mind seriously” [(1), p. 1].

^3^Thus in line with this, Graham (1) argues that going the FP-governed way ought not to encourage one to endorse the DSM atheoretical method of classifying and characterizing mental disorders.

^4^For example, Graham (1) illustrates the latter point vividly by reminding us that, “an addict … does not possess a broken brain” (p. 179). The reason this can be so, Graham (1) claims, is because, “The brain, in general, is not hard-wired for personal prudence. Neural activity may systematically underwrite unwise behaviors without exemplifying a breakdown or something wrong or damaged in its wetware or machinery” (p. 178).

^5^In pressing for this future vision of mental health as wholly grounded in science, Murphy (2) laments that much contemporary psychiatry actively shies away from theory (as exemplified in the avowed theory-neutrality of the DSMs). Against the diagnostic descriptivism of the DSMs, he maintains that psychiatry must aim to find out how things “really are” with mental disorders and what underlies them. The only way psychiatry can do this, and thus secure its future, would be to take advantage of and actively contribute to developments in the cognitive sciences.

^6^A psychiatry cast in the scientific image must assume that, “what counts as normal human nature is decided by a variety of disciplines that comprise the cognitive and biological sciences” [(2), p. 11].

^7^Murphy (2) is certainly right that there is a real and urgent need for psychiatry to address the issues that lie at the heart of the debate between FP and SI views. For the troubling fact is that, “psychiatry as it stands is not a particularly mature or successful enterprise” [(2), p. 10].

^8^Murphy’s (2) defense is admittedly qualified because he admits there are limits to our understanding when it comes to naturalizing and mechanizing central reasoning processes such that it may turn out that a proper scientific understanding of the latter might never be attainable.

^9^Such a merger, he holds, is “necessary to develop the broadest and most fertile approach to understanding psychopathology” [(2), p. 12]. In saying this, he does not promote a crude reductionism. He does not assume that neuropsychology offers molecular explanations or that its explanations are somehow more fundamental. Nevertheless, he holds, neuropsychological explanations have a privileged status: they provide a special understanding that affords unique possibilities for intervening upon and treating mental disorders.

^10^Thus, in a forthcoming paper, Murphy writes, “The question whether science makes use of representational systems isn’t really open to doubt any longer: many areas of psychology and neuroscience take for granted the existence of semantic interpretations of internal states of some cognitive system. The assumption that inputs and outputs to and from components of the brain represent distal features of the world has been part of neuroscience since the nineteenth century. What is open to doubt is whether representation, as used in the sciences of mind, has the properties that philosophers have found in intentional content, as presupposed by folk psychology. I am not taking a stand on that … ” (Murphy D. *Brains and Beliefs* (Unpublished)).

^11^Gerrans (3) follows Murphy’s lead of treating the SI view as best seen through the lens of a “minimalist cognitivism” (p. 18). Like Murphy, he sees that the future of mental health resides with brain sciences of the cognitive variety. He too regards psychiatry “as *a branch of cognitive neuroscience* by employing cognitive models that do not abstract away from, but are sensitive to, details of neural implementation” [(3), p. 37].

^12^On this vision, “personhood is a cognitive phenomenon constituted by the fact that personal-level phenomena, such as feelings, beliefs, emotions and desires arise at the highest levels of a cognitive processing hierarchy whose nature can be described and explained” [(3), p. 21]. Human cognition is thus “a complex hierarchy of computational processes performed by neural circuitry” [(3), p. 30].

^13^Motivating this proposal, with a Parthian shot at the perceived limits of the FP view, Gerrans (3) stresses that, “collecting and collating correlations between neural, phenomenological and cognitive properties of the delusional mind is useful but we need a theoretical approach that *fits all this information together*” (p. 14). It is here that we meet the idea that the tools of cognitive neuroscience are uniquely well suited to integrating “evidence from different disciplines about the way the mind configures itself in response to incoming information according to the way neural mechanisms influence cognitive processing” [(3), p. 14].

^14^There is great potential for confusion and conflation about just what the FP view and cognitive theory might, respectively, have to offer in terms of deeper explanations. As Section “Folk Psychology Rules” made clear, the FP view allows that we can go to a different level of description in order to get deeper explanations of mental disorders. Remarkably, in some places, Gerrans (3) talks in ways that suggest cognitive theory is wholly at peace with the FP view’s suggestion that underlying neural mechanisms only ever explain by describing implementing mechanisms of cognitive phenomena. As he writes: “It is normal practice *to explain phenomena* such as amnesia or macular degeneration in terms of the way neural circuits *implement the cognitive processes* involved in memory or perception. This suggests that the way to explain psychology and phenomenology in terms of neurobiology is via a cognitive theory” [(3), p. 15, emphasis added]. If this were the whole story, it would be hard to distinguish what cognitive theory could offer that is really different from what the FP view offers. Yet, there are reasons to think this is neither the whole story about what cognitive theory has to offer nor the right one. In an unpublished paper, Murphy (forthcoming) upbraids Gerrans for talking about explanations of mental disorders by appeal to implementation mechanisms. By Murphy’s lights, such talk is just an unfortunate hangover of the philosophical tendency to mix up analytic functionalism with cognitive psychology.

^15^For example, elsewhere, Gerrans speaks of the “*necessary* role of cognitive theory in linking the neurobiological and phenomenological levels of explanation” [(3), p. 18, emphasis added]. Methodologically speaking, it is strange that Gerrans (3) makes appeal to such general and wholesale philosophical justifications, for when pinning his philosophical colors to the mast he clearly tells us that: “*Murphy is right*. Our best understanding of the mind comes from understanding cognitive architecture. However that argument *cannot be established a priori for all mental phenomena*. The best we can do is construct, revise and, ultimately, unify case-by-case explanations” [(3), p. 14, emphases added]. This fits better with his more retail defenses of cognitive theory, such as when he claims that the “cognitive theory of visuo-motor control embedded in the overall architecture of cognitive control … is *required to explain* why high levels of activity in these regions produce loss of a sense of agency” [(3), p. 18, emphasis added], or when he tells us that “schizophrenic symptoms *can only be explained* in representational terms” [(3), p. 18].

^16^A CIT, intentionality-in-mechanism vision of the cognitive, is clearly in tune with the idea that, “The whole thrust of cognitive science is that there are sub-personal contents and sub-personal operations that are truly cognitive in the sense that these operations can be properly *explained only in terms of these contents*” [(18), p. 27, emphasis added].

^17^In the text surrounding this claim, Gerrans (3) makes clear that he is drawing on assumptions that predictive coding accounts of perception have made popular to support the idea that visual systems and scientists “do essentially the same thing.” Predictive coding accounts understand cognition as a matter of making active inferences in continuous effort to minimize prediction error. But whether we should think of visual systems as really making contentful inferences at all, and whether if they do, they do so in anything like the way that scientists do, are highly contentious topics of current debate [for reasons why we ought to prefer a non-contentful reading of predictive processing, see Ref. (20, 21)]. For this reason, it might seem safer for cognitivists to advance a weaker claim about what makes these phenomena essentially the same. It might be argued that scientists and visual systems are essentially alike because they both use representations even though visual systems use different kinds of representation than scientists do. The idea here is that there is no requirement that visual systems and scientists need to operate with the same kinds of content in order to qualify as representational systems. While it is technically correct to go this way it raises afresh the question of what unifies and intelligibly relates these two cognitive phenomena if not the fact that they both involve manipulations of content of the same kind. The point is that without full details, it is far from clear why we should accept that vision and scientific theorizing – which appear to be quite disparate cognitive activities – are essentially alike.

^18^When thinking about what might be leftover in such a subtraction, it is useful to consider Gerrans’s (3) claim that, “different cognitive processes have *different computational properties* that enable them to meet their congruence condition. These *properties provide the intentional structure of representations* produced by different cognitive processes. For example, the representations produced by the visual system are 3D coloured scenes derived by processing spectral and luminance information” [(3), p. 105, emphases added]. However, once no correctness conditions are in play, it is not clear in what sense the residual structures ought to be thought to bear representational content or even what it means to say that they do (22).

^19^Enactivists, of course, encourage multi-stranded investigations, involving explanations that are pitched at various “levels” and “scales.” Gerrans (3) acknowledges this. Taking the case of vision as a prime example, he emphasizes the need for theories that seek to simultaneously investigate different levels of cognitive activity and how they integrate. Yet here he notes, “Even enactive theorists of vision who disagree with Marrians nonetheless debate with them about the causal relevance of mechanisms at different levels” [(3), p. 43].

^20^Crucially, however, Murphy (2) insists that it is important to “distinguish between supplementing and replacing the medical model” (p. 367).

^21^Narrative therapy is, for example, neither scientifically focused nor scientifically based. It uses special techniques in order to provide the tools for empowering people, enabling them to exercise their agency in wider and more positive ways. Narrative therapy, although very much in the mould of “talking cures,” is thus unlike more familiar psychoanalytic approaches to therapy in that it does not seek to divine and understand past causes of current trauma. Nevertheless, there seems no reason to discount narrative therapy as a bone fide therapy given that it is has been used successfully to help people deal better with a wide range of psychiatric and traumatic conditions, including asthma, anorexia, bulimia, and depression.

^22^Hutto DD, Gallagher S. Re-authoring narrative therapy: opening the way for future developments. *Philos Psychiatr Psychol* (Forthcoming).

^23^Whether FP should be understood as a narrative practice as opposed to, and distinct from, a theory of mind remains a controversial matter of dispute in the literature. It would take too much space to attempt to settle the issue or detail all the consequences of going one way rather than the other, in this paper. Extended arguments for treating FP as a narrative practice that does not reduce to theory can be found in Ref. (23, 25–28, Hutto DD, McGivern P. Updating the story of mental time travel: narrating and engaging with our possible pasts and futures. In: Altshuler R, Sigrist MJ, editors. *Time and the Philosophy of Action*. London: Routledge (Forthcoming)).
